# TrAnnoScope: A Modular Snakemake Pipeline for Full-Length Transcriptome Analysis and Functional Annotation

**DOI:** 10.3390/genes15121547

**Published:** 2024-11-29

**Authors:** Aysevil Pektas, Frank Panitz, Bo Thomsen

**Affiliations:** 1 Department of Molecular Biology and Genetics, Aarhus University, 8000 Aarhus, Denmark; aysevilpektas@mbg.au.dk (A.P.); frank.panitz@luke.fi (F.P.); 2 Applied Statistical Methods, Natural Resources Institute Finland (Luke), 20520 Turku, Finland

**Keywords:** RNA-Seq, reproducible pipeline, high-performance computing (HPC), transcriptome analysis, functional annotation, Iso-Seq, snakemake, long-read sequencing

## Abstract

**Background/Objectives**: Transcriptome assembly and functional annotation are essential in understanding gene expression and biological function. Nevertheless, many existing pipelines lack the flexibility to integrate both short- and long-read sequencing data or fail to provide a complete, customizable workflow for transcriptome analysis, particularly for non-model organisms. **Methods**: We present TrAnnoScope, a transcriptome analysis pipeline designed to process Illumina short-read and PacBio long-read data. The pipeline provides a complete, customizable workflow to generate high-quality, full-length (FL) transcripts with broad functional annotation. Its modular design allows users to adapt specific analysis steps for other sequencing platforms or data types. The pipeline encompasses steps from quality control to functional annotation, employing tools and established databases such as SwissProt, Pfam, Gene Ontology (GO), the Kyoto Encyclopedia of Genes and Genomes (KEGG), and Eukaryotic Orthologous Groups (KOG). As a case study, TrAnnoScope was applied to RNA-Seq and Iso-Seq data from zebra finch brain, ovary, and testis tissue. **Results**: The zebra finch transcriptome generated by TrAnnoScope from the brain, ovary, and testis tissue demonstrated strong alignment with the reference genome (99.63%), and it was found that 93.95% of the matched protein sequences in the zebra finch proteome were captured as nearly complete. Functional annotation provided matches to known protein databases and assigned relevant functional terms to the majority of the transcripts. **Conclusions**: TrAnnoScope successfully integrates short and long sequencing technologies to generate transcriptomes with minimal user input. Its modularity and ease of use make it a valuable tool for researchers analyzing complex datasets, particularly for non-model organisms.

## 1. Introduction

RNA sequencing (RNA-Seq) has become a powerful tool for the detection of novel transcripts, understanding gene expression, cataloging protein-coding genes, and revealing the biological functions of genes [1,2,3]. Additionally, RNA-Seq has enabled the study of non-model organisms, without the need for a reference genome, through de novo transcriptome analysis [4]. However, there are several challenges associated with RNA-Seq analysis, such as sequencing errors and fragmentation resulting from technological limitations, in addition to issues such as repetitive regions and overlapping genes due to transcriptome complexity [2,4].

Short-read technologies have lower error rates and provide higher coverage than long-read sequencing technologies. Nevertheless, transcriptomes generated exclusively from short reads often suffer from fragmentation and incomplete transcript reconstruction due to the erroneous computational predictions of isoforms. In contrast, long-read sequencing technologies can capture FL transcripts and resolve the isoform complexity; however, they retain a higher error rate and lower throughput. Accurate transcriptome assembly is crucial for downstream analyses, including functional genomics, gene discovery, and the elucidation of complex biological processes [4]. Hybrid approaches that leverage the strengths of both short- and long-read technologies can overcome the weaknesses of each technology and improve the transcriptome coverage and accuracy to obtain known and novel transcripts [2,5]. In particular, a hybrid approach that uses short reads for the error correction of long reads can significantly reduce the error rate in the long reads, enhancing the accuracy of the transcriptome for downstream analysis while preserving the full-length structure of the transcripts [6,7,8].

Transcriptome generation and annotation are challenging because of the complexity of the procedures, the need to select appropriate tools, and the significant computational resources required [3]. Several RNA-Seq pipelines offer an interconnected collection of tools designed to automate the process, such as RNAflow [9] and RASflow [10], which primarily focus on differential expression analysis, while others, such as TransXpress [11], TransPi [12], and Pincho [13], focus on de novo transcriptome assembly and functional annotation. However, these tools depend on short-read sequencing for analysis.

Several toolkits, such as Functional IsoTranscriptomics Analysis (FIT) [14], IsoTools [15], TAGET [16], and nf-core/isoseq [17], utilize the properties of long-read sequencing technologies for transcriptome analysis. However, they are primarily designed to function with reference annotations, and, currently, Trans2Express [18] is the only reproducible protocol for non-model organisms. It enables de novo hybrid transcriptome assembly using both the Illumina and Oxford Nanopore Technologies (ONT) platforms, aiming to recover a single transcript per gene for transcriptome characterization and gene expression analysis. However, this approach may lead to the loss of important information relating to alternative splicing and isoform diversity and limit the detection of novel transcripts or isoforms, especially in the less-studied regions of a transcriptome; a more complete approach that captures multiple isoforms is essential in fully elucidating the functional potential of genes [19]. Furthermore, Trans2Express offers limited flexibility, restricting users from selecting and combining tools within the pipeline to meet their specific research objectives.

Here, we present TrAnnoScope, a comprehensive FL transcriptome and annotation pipeline that integrates Illumina short-read and PacBio long-read data through a number of key steps, including data preprocessing, long-read error correction, contamination removal, quality assessment, and functional annotation. The pipeline was designed to improve the transcriptome accuracy and completeness by leveraging the strengths of long reads enhanced by short reads and with thorough preprocessing without the need for reference annotation. By using high-accuracy Illumina short reads to correct errors in long reads, TrAnnoScope reduces the error rate while preserving the FL structure of transcripts. The purpose of this correction step is to minimize errors, resolve the isoform complexity, and enhance the transcriptome completeness, enabling more accurate downstream analyses, such as gene discovery and functional annotation. TrAnnoScope is highly modular, allowing users to customize their workflows and integrate different components to suit their research goals. In addition, it supports parallel execution and cluster computing, enabling the faster processing of larger datasets. It is sufficiently versatile to be used in many research areas, from gene discovery and transcriptome profiling to the study of complex biological systems. As a result, TrAnnoScope provides a powerful, reproducible approach to conducting efficient bioinformatics analyses for large-scale transcriptome analyses.

## 2. Materials and Methods

### 2.1. Components of the TrAnnoScope Pipeline

**Figure 1:**
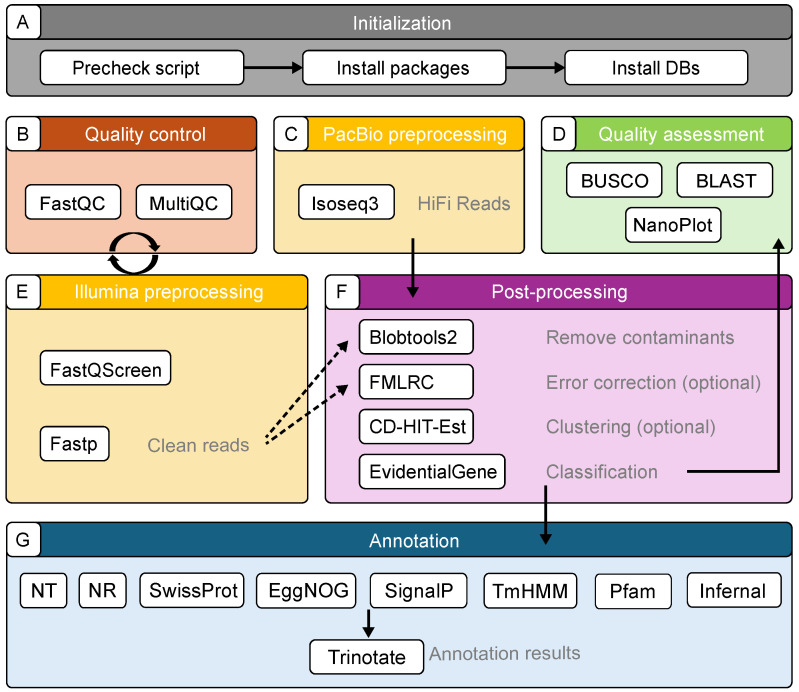
Overview of the TrAnnoScope pipeline, consisting of the following steps: (**A**) initialization, (**B**) quality control of Illumina reads, (**C**,**E**) preprocessing of Illumina and PacBio reads, respectively; (**D**) quality assessment; (**F**) post-processing, including the removal of contaminants, error correction, and cluster and classification steps, and (**G**) annotation. Arrows in the figure indicate the inputs for each corresponding step, showing the flow of data and dependencies between steps.

We implemented our pipeline using Snakemake owing to its simplicity and ability to automate complex workflows while managing dependencies [20]. The TrAnnoScope pipeline consists of several modules, starting with a Python v3.10.14 script (Figure 1A) that simplifies the setup by installing the necessary dependencies and databases for TrAnnoScope, allowing users to install only the components that are essential for their needs. Following this, the pipeline includes the quality control of Illumina reads (Figure 1B), preprocessing of Illumina reads (Figure 1E), preprocessing of PacBio reads (Figure 1C), contamination removal, error correction using Illumina reads, isoform clustering and classification (Figure 1F), quality assessment (Figure 1D), and annotation (Figure 1G). A configuration file is provided for users to customize the tool parameters according to their needs. To address the time-intensive steps, such as contamination removal and annotation, TrAnnoScope supports parallel execution by dividing the input files for faster processing. Detailed instructions are available on the TrAnnoScope GitHub page (https://github.com/aysevllpkts/TrAnnoScope, accessed on 27 November 2024).

To fully utilize TrAnnoScope, users must provide both PacBio long reads and Illumina short reads, with the latter used to improve the long-read data through error correction. However, the pipeline also supports the running of individual steps independently, enabling users to tailor the workflows to their specific needs. For instance, users can choose to run only the quality control and preprocessing modules for Illumina reads to obtain clean short reads or execute the preprocessing, clustering and classification, quality assessment, and annotation modules with only PacBio reads to obtain full-length annotated transcripts; alternatively, although TrAnnoScope does not perform transcript assembly and annotation using only short reads, it can be used to annotate and assess the quality of an existing short-read transcriptome assembly or other transcriptome assemblies created from different data types through the quality assessment and annotation steps. Furthermore, users can customize the annotation process by selecting from the databases provided by the pipeline or incorporating their own databases. This modularity ensures that TrAnnoScope can be adapted to a wide range of transcriptome analysis tasks and data types.

Additionally, TrAnnoScope offers flexibility through its modular design. Users can customize the software parameters by modifying the configuration file, allowing for adjustments to various pipeline components. For example, users can create a stricter transcriptome by applying more stringent filtering criteria, such using a lower similarity percentage for clustering in the classification step or increasing the threshold for the number of reads in consensus sequences in the preprocessing of the PacBio reads step, which helps to remove low-quality and low-coverage sequences. Alternatively, users can obtain a broader transcriptome by applying more relaxed options to include a wider range of transcripts.

### 2.2. Pipeline Implementation

#### 2.2.1. Quality Control and Preprocessing of Illumina Reads

The first module of the TrAnnoScope pipeline focuses on performing the quality control of Illumina short reads, which is an essential step in ensuring data reliability. FastQC [21] is used to generate quality reports for individual samples, and MultiQC [22] combines these reports into a detailed summary of all Illumina datasets.

To detect potential contamination, FastQScreen [23] maps the reads against a set of reference databases. TrAnnoScope allows users to use the default FastQScreen Genome database or create a custom database to filter out undesired sequences, such as species-specific mitochondrial RNA (mtRNA) or ribosomal RNA (rRNA) sequences. For adapter trimming and quality filtering, fastp [24] is employed due to its speed, low memory usage, and detailed quality reports. Additionally, it detects and removes poly-G tails from Illumina NextSeq/NovaSeq data. Ensuring high-quality reads, such as via contamination removal and error correction, is crucial for downstream analysis, as poor-quality reads can lead to inaccurate results. A final quality control step is performed for the preprocessed reads using FastQC and MultiQC to confirm that they are suitable for downstream analysis.

#### 2.2.2. Preprocessing of PacBio Reads

The Isoseq3 package [25] is used to preprocess PacBio reads, which is essential in converting raw sequencing data into high-quality FL transcripts. This module begins by converting subreads into circular consensus sequences (CCSs), which are self-corrected sequences, using the ccs to ensure higher accuracy in the downstream analysis. The lima is then used to demultiplex and trim the primer sequences from the CCS reads to obtain FL reads. Further, isoseq3 refine removes chimeric sequences, which can originate from multiple transcripts, and retains only reads with poly-A tails, producing FL non-chimeric (FLNC) reads that improve the read quality. Isoseq3 cluster/cluster2 groups the reads based on their sequence similarity to identify unique transcripts and their isoforms, offering insights into alternative splicing and transcript diversity, both of which are crucial in understanding gene expression dynamics. This preprocessing step currently applies only to PacBio reads. However, if the user has Nanopore reads in the FASTA format, they can still be used for the subsequent steps of the pipeline.

#### 2.2.3. Contamination Removal

Contaminants in RNA-Seq data can significantly impact the quality and accuracy of the results, leading to biased gene expression and the incorrect identification of splice variants. These contaminants can be introduced at various stages of the RNA-Seq process [26,27].

To address this, the TrAnnoScope pipeline uses Blobtools2 [28] to remove microbial and cross-contamination from long reads. Blobtools2 analyzes, visualizes, and filters assemblies based on the GC content, coverage, and taxonomic information. This tool is especially useful for de novo data, helping to identify and remove contaminants, thereby improving the quality of assemblies. The input files for Blobtools2 are generated using Bowtie2 [29] for coverage data, Benchmarking Universal Single-Copy Orthologs (BUSCO) for taxonomic classification [30], and BLAST [31] against the National Center for Biotechnology Information (NCBI) nucleotide (NT) database for taxonomic information.

#### 2.2.4. Error Correction

Error correction can be beneficial in improving the accuracy of long reads, which typically have higher error rates than short reads [8]. In TrAnnoScope, FMLRC [32] is available as an optional step for the correction of errors in long reads by leveraging complementary Illumina reads. FMLRC utilizes a multi-string Burrows–Wheeler transform and FM index to retrieve k-mer frequencies and construct de Bruijn graphs from short reads. It performs two passes with short and long k-mer values to correct unsupported regions in long reads, resulting in a more thorough correction process. Owing to its efficiency and accuracy, FMLRC is a robust choice for error correction [8].

#### 2.2.5. Clustering and Classification

To eliminate redundancy and reduce the complexity of the transcript data, TrAnnoScope employs two tools for clustering and classification: CD-HIT-Est [33], which is provided as an optional tool, and EvidentialGene [34]. CD-HIT-Est clusters transcripts based on their sequence similarity to remove redundancy within each sample, whereas EvidentialGene classifies the transcripts as primary and alternate forms based on their quality and potential function across the combined dataset and predicts the protein sequences.

#### 2.2.6. Quality Assessment

TrAnnoScope includes a quality assessment step that utilizes several tools to evaluate the transcriptome. NanoPlot [35] generates descriptive statistics, such as the mean, median, and N50 values, providing a clear overview of the transcript continuity. BUSCO [30] further assesses the transcriptome completeness against a user-defined lineage, ensuring the presence of elements for the organism of interest. Additionally, the transcriptome quality is evaluated by comparing the number of FL or nearly FL transcripts against known protein databases, using an approach similar to the Trinity method of counting FL transcripts [36]. However, we implemented an in-house Bash script that calculates the percentage of high-coverage proteins present in the transcriptome compared to SwissProt by default or a user-defined custom database for closely related organisms. Together, these methods provide a detailed and accurate measure of the transcriptome quality.

#### 2.2.7. Annotation

The functional annotation of the transcriptome is performed using Trinotate [37], an advanced annotation suite created for the automated functional annotation of transcriptomes. Trinotate integrates multiple sequence databases, including Pfam [38], SwissProt [39], SignalP [40], TMHMM [41], EggNOG [42], and Infernal [43], to provide broad annotation. Additionally, TrAnnoScope provides homology searches against the NCBI (non-redundant protein) NR and NT databases. Users can select the databases that they wish to use for annotation in the configuration file. However, they must manually download and prepare the necessary files for the NT and NR databases prior to the annotation process. To facilitate this, bash scripts to automate the downloading and indexing of the databases are provided.

To perform homology and protein domain searches against the zebra finch transcriptome, various tools were employed. For homology searches against the SwissProt and NR databases, DIAMOND blastx/blastp were used with the following parameters: -max-target-seqs 1 -evalue 1e-5 -outfmt 6 std stitle. A protein domain search was performed using hmmsearch with the –noali parameter against the Pfam database. For signal peptide prediction, signalp6 was run with the –format none –organism euk –mode fast parameters. Transmembrane domain predictions were obtained using tmhmm with the –short parameter. Functional annotation against the EggNOG database was performed using emapper.py with the default parameters. To identify non-coding RNAs, the cmscan from Infernal was utilized with the parameters -Z 5 –cut_ga –rfam –nohmmonly –fmt 2. Finally, for homology searches against the NT database, blastn was used with the parameters -max_hsps 1 -max_target_seqs 1 -evalue 1e-5 -outfmt “6 std stitle”. All results were combined using Trinotate to generate the final annotation file with the default parameters.

To accelerate the annotation process, we implemented a strategy that splits the input files into user-defined chunks, enabling the parallel execution of homology searches. This approach significantly reduces the time required to annotate large datasets. After completing the homology searches, the results were parsed using modified Trinotate helper scripts to generate detailed annotation files enriched with GO [44] terms from the SwissProt, Pfam, and EggNOG databases. An updated version of the extract_GO_assignments_from_Trinotate_xls_updated.pl script was used, which extends the functionality to include GO terms from EggNOG alongside those from SwissProt and Pfam. To provide a concise representation of the GO categories, the Trinotate_GO_to_SLIM.pl script was employed to map the GO terms to their corresponding GO slim categories. KEGG [45] pathways and KOG [46] classifications were also derived from the homology searches performed against the EggNOG database, with distribution plots generated using in-house R scripts. Additionally, species distribution plots were created based on the Blastx homology search results against the NCBI NR database using custom R scripts.

#### 2.2.8. Data Selection

To demonstrate the functionality and versatility of our pipeline, we processed publicly available RNA-Seq reads from the zebra finch (*Taeniopygia guttata*), an avian model used to study the neural mechanisms of local learning and social behavior [47]. This species is notable for its complex vocalization, ease of breeding in captivity, and pronounced sexual dimorphism, making it a valuable model in understanding vocal learning and its implications for human speech and language development [47,48].

RNA-Seq data were obtained from the NCBI Sequence Read Archive [49] (SRR8551559, SRR8551563, SRR8551565, SRR8551567, SRR8551558, SRR8551562, SRR8551564, and SRR8551566). This dataset includes Illumina NextSeq 500 paired-end reads (2 × 76 bp) and long-read sequences generated via PacBio SMRT Sequel from various tissue types, including the brain, ovary, and testis (Table S1). These complementary data types provided an ideal scenario for the evaluation of the ability of the pipeline to integrate and interpret both short and long reads for a comprehensive transcriptome assembly. TrAnnoScope was executed on the GenomeDK cluster using SLURM for all analysis steps. Detailed information about the outputs and runtime is provided in Supplementary File S3.

#### 2.2.9. Mapping to the Zebra Finch Reference Genome

The final transcriptome generated by the TrAnnoScope pipeline was mapped to the current zebra finch reference genome (RefSeq: GCF_003957565.2) using the minimap2 with -ax splice –secondary=no -C5 parameters. Alignment statistics were obtained using the samtools flagstats option. To extract aligned transcripts associated with zebra finch genes, Bedtools v2.30.0 was used with the parameters intersect -wao -bed. The genes assigned to each transcript were integrated into the Trinotate annotation file.

## 3. Results and Discussion

In this study, we applied TrAnnoScope to RNA-Seq data from the zebra finch (*T. guttata*) to evaluate its effectiveness in transcriptome assembly and functional annotation. The dataset included Illumina reads and PacBio long-read sequences from brain, ovary, and testis tissue. Our primary objective was to assess the ability of the pipeline to integrate and interpret these complementary data types to achieve a comprehensive transcriptome assembly. This section details the results obtained, focusing on the key findings related to preprocessing, contamination removal, error correction, clustering and classification, quality assessment, and functional annotation.

In the preprocessing step for Illumina reads, we evaluated the read quality before and after data processing using FastQC, with the quality metrics compiled into a single report for concise visualization via MultiQC. Contaminants, including rRNA, mtRNA, and other potential contaminants, were removed using FastQScreen. This process involved hits against the LSU_Ref and SSU_Ref Silva databases v.138 [50], the zebra finch mitochondrial genome (NCBI Reference Sequence: NC_007897.1), and the FastQScreen database of vectors, adapters, and GRCm38 rRNA. For each sample, only minor hits were detected in the rRNA databases, primarily mitochondrial reads from the zebra finch (~5%); approximately 95% of the reads had no hits (Figure S1). Reads that did not map to these databases (no hits) were retained for downstream analyses. Adapter sequences and low-quality bases were trimmed using fastp. Table 1 presents the preprocessing statistics for each Illumina sample. Following preprocessing, the number of retained high-quality reads ranged from 27,446,223 to 33,201,568 per sample, ensuring robust data for the downstream assembly.

The initial preprocessing of the PacBio raw data (subreads) followed the Isoseq3 package for each sample. CCSs were generated using the default minimum number of subreads (default: 3). FL transcripts were identified, and primers were removed using lima with –peek-guess, while isoseq3 refine was used to remove poly-A tails and artificial concatemers to obtain FLNC reads. High-quality FL consensus sequences were obtained using the isoseq3 cluster2 with the –singletons parameter. By default, isoseq3 cluster2 retains isoforms that are represented by at least two FLNC reads. To capture rare but potentially significant isoforms, the --singletons option was employed for this analysis to include these single-read isoforms in the consensus sequences.

To eliminate potential contamination from PacBio reads, BlobTools2 was employed to retain only vertebrate sequences and no hits, the latter being sequences not assigned to any taxonomic group, for further analysis. Except for the ovary_2 sample, which contained hits from the *Annelia* phylum, all other samples contained only vertebrate sequences and no hits (Figure S2). Additionally, rRNA and mitochondrial fragments were identified and removed by aligning the reads against the NCBI nucleotide database (NT, retrieved on 9 February 2024) using Blastn. High-quality, clean FL reads from each sample were corrected using FLMRC with the default parameters. The error correction step is provided as an option for users. With the advancements in sequencing technologies, the accuracy of long reads has been gradually increasing. The error rate of Nanopore sequencing has improved from approximately 64% for R7 to approximately 84–95% for R9.4 [51]. In contrast, the PacBio platform, utilizing the CCS approach, achieves greater than 99% consensus accuracy [52,53]; however, systematic errors can still persist, especially in homopolymeric regions [54,55]. Depending on the accuracy of the sequencing platform utilized, users can proceed with downstream analysis without an additional error correction step. In our case study, the error correction step was applied to the long-read transcriptome data, resulting in significant improvements in the BUSCO scores. When comparing the error-corrected and non-error-corrected long reads (Table S2), the BUSCO results showed an increase in complete and duplicated BUSCOs and a decrease in single, fragmented, and missing BUSCOs. The statistical significance of these differences was confirmed by the paired t-test results (Table S3, Figure S3), highlighting the importance of the error correction step in our process. The significant decrease in fragmented BUSCOs suggests that error correction helps to resolve sequences previously interpreted as partial models due to sequencing errors. Additionally, the reduction in missing BUSCOs indicates that sequences that were previously unknown due to errors became identifiable after correction. These improvements contributed to the overall increase in complete BUSCOs. Overall, these results highlight the importance of error correction in enhancing the completeness and accuracy of transcriptome data. While this step is optional in TrAnnoScope, our findings strongly support its inclusion when processing Iso-Seq data. This finding aligns with the literature suggesting that error correction can still enhance the overall quality of transcriptomic data [53]. Table 2 shows the number of reads obtained at each preprocessing step. To eliminate redundancy, CD-HIT-Est was first employed for each sample (Table 2), and, subsequently, EvidentialGene was used for the combined sample for the further classification of the mRNA reads and predicted protein sequences (Table 3).

**Figure 2:**
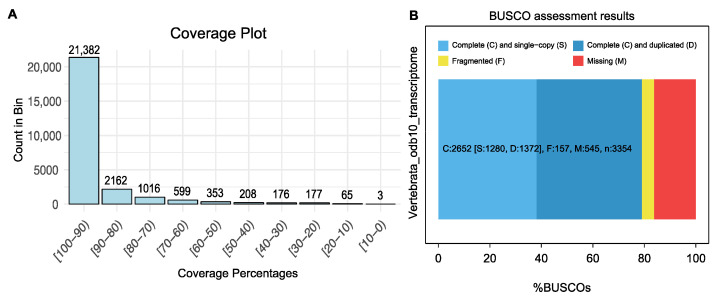
(**A**) Bar plot for significant hits from BLASTP (e-value= 1 × 10^−20^) against the zebra finch proteome. (**B**) BUSCO assessment of the transcriptome against vertebrata_odb10, showing the distribution of complete (C), single-copy (S), duplicated (D), fragmented (F), and missing (M) BUSCOs to evaluate the transcriptome completeness and quality.

After EvidentialGene, 39,984 transcripts were obtained, with an average transcript length of 3097.7 bp, a median of 2794 bp, and an N50 of 4108 bp, indicating the robustness of the assembly and the inclusion of long, high-quality transcripts (Table 3). Among the 39,984 predicted proteins, the average protein length was 398.2 amino acids, with a median of 271 amino acids and an N50 of 597 amino acids. Notably, 86.7% of the transcripts were classified as complete proteins, further demonstrating the effectiveness of the pipeline in generating FL sequences. For FL representation analysis, a total of 26,141 transcripts matched the zebra finch protein sequences (GCF_003957565.2), with an e-value threshold of 1 × 10^−20^. Of these, 24,560 (93.95%) transcripts presented as nearly FL (>70% coverage) relative to the zebra finch reference protein sequences. Among these, 10,575 transcripts were classified as FL transcripts with 100% coverage (Figure 2A, Table S4).

The BUSCO assessment results demonstrated the completeness of the transcriptome based on the presence of BUSCOs from the vertebrate lineage (Figure 2B). The analysis revealed that the FL transcripts matched 83.8% of the single-copy orthologs in the BUSCOs, comprising 79.1% complete orthologs and 4.7% fragmented orthologs out of a total of 3354 orthologs. Additionally, 16.2% of the orthologs were classified as missing (Table 3). While this BUSCO result indicates a reasonably high level of completeness, it also highlights potential limitations in the transcriptome assembly. The observed BUSCO completeness score can be attributed to the limited sampling of only three specific tissue types. BUSCO scores assess the presence of conserved, single-copy orthologs expected in a comprehensive transcriptome. By focusing on a smaller number of tissue types, only a subset of the actively expressed transcripts was captured, which reduced the overall BUSCO score. This result does not indicate poor data quality but reflects the targeted nature of the sampling strategy [56]. To improve the completeness of the transcriptome, future studies could incorporate additional tissue samples to capture a broader range of gene expression. Despite the limitations, the BUSCO results, combined with the descriptive statistics, underscore the ability of the pipeline to accurately obtain transcriptomes from Illumina and PacBio data, providing a reliable foundation for subsequent functional analyses.

The functional annotation of the transcripts was conducted using a wide range of databases, using Trinotate with the default parameters (e-value 1 × 10^−5^), providing valuable insights into the roles and characteristics of the predicted proteins (Table 4). The annotation revealed broad coverage across multiple databases, enhancing our confidence in the functional assignments. Supplementary File S4 contains the annotation results for the transcripts, providing the functional annotations and sequence homologies identified through the pipeline. Out of the 39,984 transcripts, 70.7% (28,274) had significant hits against the UniProt/SwissProt database using Blastx, while 62.7% (25,051) were confirmed through Blastp searches. Domain-based searches using Pfam identified conserved protein domains in 59.2% (23,689) of the transcripts, highlighting their protein-coding potential.

**Figure 3:**
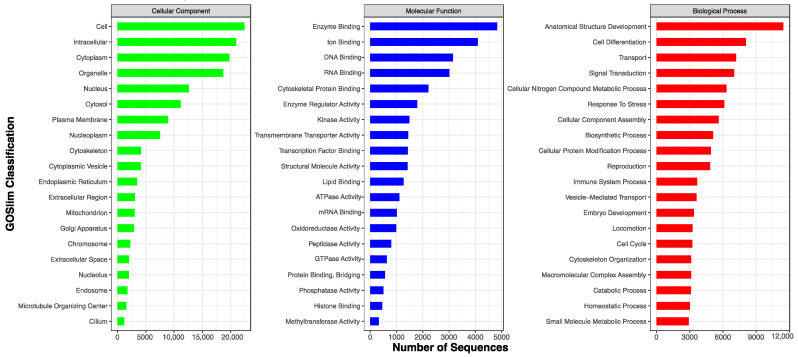
Distribution of top 20 Gene Ontology terms for the cellular components, molecular functions, and biological processes of zebra finch.

**Figure 4:**
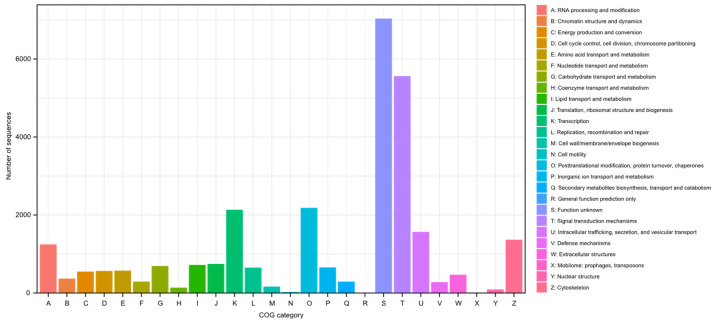
KOG classification of zebra finch.

**Figure 5:**
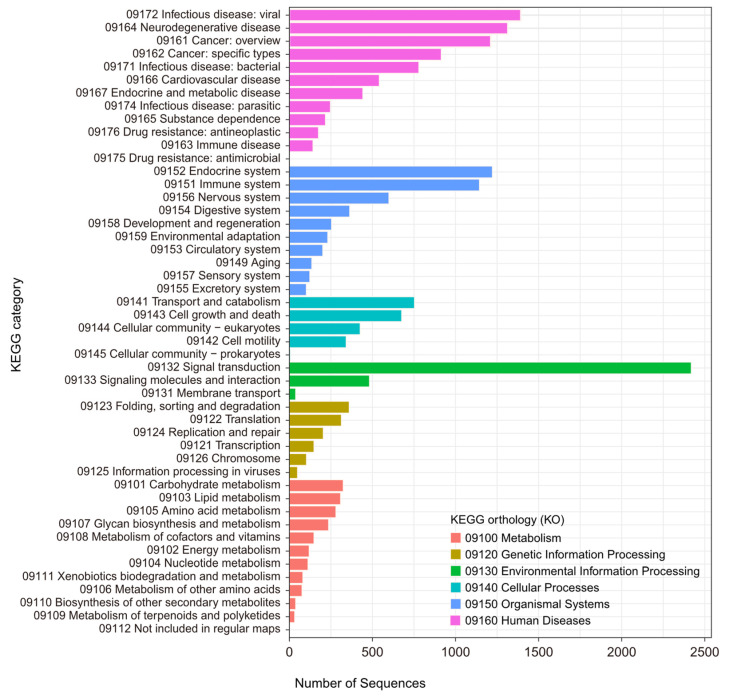
KEGG classification of zebra finch.

GO terms were assigned to 71.0% (28,399) of the transcripts based on homology searches against the SwissProt, Pfam, and EggNOG databases. These GO terms were further categorized into biological processes, molecular functions, and cellular components, providing an overview of the functional landscape (Figure 3). Additionally, KOG classifications were identified for 66.0% (26,409) of the transcripts through homology searches against the EggNOG database, offering insights into their evolutionary relationships and functional roles (Figure 4). The KEGG pathway analysis annotated 65.3% (26,097) of the transcripts, linking them to various metabolic and signaling pathways derived from the EggNOG annotations (Figure 5).

Structural and localization predictions identified transmembrane domains in 17.9% (7155) of the transcripts, as predicted using TMHMM, and signal peptides in 5.8% (2305), as predicted using SignalP v6.0. A small portion of the transcripts (0.4%, 169) were annotated as non-coding RNAs using Infernal.

Further annotation against the NR database using Blastx produced hits for 82.7% (33,049) of the transcripts, while Blastp hits were obtained for 70.8% (28,303). Nearly all transcripts (99.6%, 39,827) matched in the NT database through Blastn. These broad annotation results provide a rich resource for downstream biological analyses.

**Figure 6:**
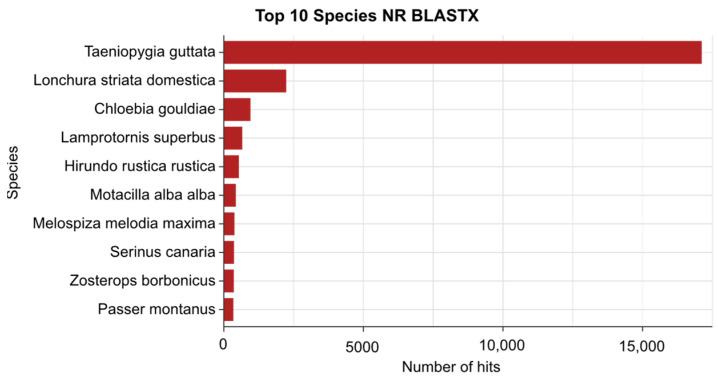
Top 10 species distribution of the transcript sequences of the zebra finch against the NR database.

The species distribution of the transcripts obtained from the TrAnnoScope pipeline underscores the effectiveness of our approach in the functional annotation of zebra finch data (Figure 6). The Blastx homology search against the NR database revealed that a significant number of hits (17,121) were assigned to the zebra finch, indicating strong alignment between our assembled transcripts and existing annotations. This result highlights the robustness of the TrAnnoScope pipeline in processing RNA-Seq data and achieving meaningful annotations that are crucial for downstream analyses. Furthermore, the presence of additional hits to closely related species, such as the society finch (*Lonchura striata domestica*), Gouldian finch (*Chloebia gouldiae*), and canary (*Serinus canaria*), can be attributed to the high degree of genetic similarity among these species. This observation reinforces the notion that functional conservation is common among closely related species, facilitating the identification of homologous genes and conserved biological functions. Moreover, the identification of transcripts that align with other birds in the Passeriformes order, such as starlings (*Lamprotornis superbus*), swallows (*Hirundo rustica rustica*), sparrows (*Melospiza melodia maxima, Passer montanus*), and Réunion grey white-eye (*Zosterops borbonicus*), reflects the shared ancestry within this diverse avian group.

The successful functional annotation of these transcripts not only validates the efficiency of the TrAnnoScope pipeline but also provides valuable insights into the evolutionary relationships among these avian species. The alignment of our data with those of the zebra finch and its relatives may contribute to a deeper understanding of the genetic basis of traits relevant to adaptation and survival in varying environments. Additionally, these findings can serve as a foundation for future studies aimed at exploring gene function, expression patterns, and evolutionary dynamics within the Passeriformes order and beyond.

To validate the transcripts generated by the TrAnnoScope pipeline and assess their biological relevance, we mapped the transcriptome to the current zebra finch genome. The alignment of our transcriptome yielded a high mapping rate of 99.63%, underscoring the accuracy and reliability of the TrAnnoScope pipeline in generating high-quality transcriptomic data.

Despite this high mapping rate, a total of 154 transcripts did not map to the genome. Among these unmapped transcripts, a significant portion (136 transcripts) exhibited notable hits in homology searches (see Supplementary File S2). This indicates that these transcripts likely represent real biological data rather than artifacts. Interestingly, 7 of these 136 transcripts aligned with sequences from previous zebra finch genome assemblies that are not present in the current genome version. This suggests that the unmapped transcripts may represent genomic regions that have been lost or altered in the latest assembly. This observation highlights the ongoing refinement of genomic resources and emphasizes the importance of considering multiple assembly versions in genomic data analysis. The rest of the unmapped 136 transcripts aligned with closely related species, particularly those within the passerine bird family. This indicates that these transcripts may possess functional relevance, potentially aligning with genes conserved across closely related passerine species and suggesting a shared heritage that may be critical in understanding the evolutionary relationships in this group. Among the remaining unmapped transcripts lacking homology search results, eight showed hits only for SignalP and TmHMM, indicating that they might encode peptides with specific targeting signals or transmembrane domains. This finding hints at their potential functional roles within cellular processes. However, the remaining ten transcripts, which yielded no significant information, raise questions about their biological significance. These transcripts could represent novel genes, warranting further exploration into their functions. Alternatively, they may be artifacts, emphasizing the need for additional validation.

While the TrAnnoScope pipeline has demonstrated its effectiveness in processing and annotating transcriptomic data, there are several areas for future improvement. One significant limitation is the current support for long-read preprocessing, which is restricted to PacBio reads. Incorporating preprocessing capabilities for ONT reads would greatly enhance the versatility of the pipeline, allowing users to leverage the strengths of both sequencing platforms for comprehensive transcriptome assembly. Additionally, providing support for differential expression analysis within the pipeline would facilitate more in-depth investigations into gene expression patterns across various conditions and tissue types. This enhancement could empower researchers to derive meaningful biological insights from their data, further expanding the utility of TrAnnoScope in the field of transcriptomics. Addressing these gaps will not only improve the overall functionality of the pipeline but also increase its appeal to a broader range of users conducting diverse transcriptomic studies.

## 4. Conclusions

In this study, we introduced TrAnnoScope, a comprehensive pipeline for transcriptome analysis and annotation that utilizes both short- and long-read data. Applying TrAnnoScope to zebra finch RNA-Seq data demonstrated its capability to generate high-quality transcripts and functional annotations across multiple databases. The pipeline efficiently processes large datasets, from quality control to final annotation, resulting in a transcriptome with significant functional insights.

TrAnnoScope is built with Snakemake, and its modular design allows for easy customization while requiring minimal programming skills, making it accessible to users with varying levels of expertise. Its parallelized steps and user-defined parameters enhance the speed and reliability of transcriptome analysis. Although some manual database preparation is necessary, the pipeline remains a valuable tool for researchers, particularly those working with non-model organisms. Future updates will focus on automating database management and expanding the preprocessing options for other platforms.

Overall, TrAnnoScope is a versatile and efficient tool for transcriptomics, providing a robust platform for transcriptome analysis.

## Figures and Tables

**Table 1:** Preprocessing step results for Illumina reads.

Step/Sample	Brain_2	Brain_5	Ovary_2	Testis_5
Raw	32,217,548	29,323,820	33,201,568	28,474,620
FastQScreen	30,396,892	28,010,526	31,965,452	27,981,280
fastp	29,777,552	27,446,223	31,089,662	27,519,646

**Table 2:** Preprocessing steps for PacBio reads.

Step/Sample	Brain_2	Brain_5	Ovary_2	Testis_5
Raw	444,968	717,758	483,419	729,821
CCS	124,615	56,773	198,608	42,832
FL	93,967	15,245	172,050	25,332
FLNC	89,017	15,103	168,336	25,095
Clustered	47,129	10,229	80,405	18,101
Contamination	46,769	9990	80,166	17,993
Error Correction	46,769	9990	80,166	17,993
CD-Hit-Est	23,636	6175	37,838	11,284

**Table 3:** Descriptive statistics of the zebra finch transcriptome obtained using TrAnnoScope.

Step/Sample	Transcriptome
Total isoforms	39,984
Mean, median, N50 transcripts	3097.7|2794|4108
Total proteins	39,984
Mean, median, N50 proteins	398.2|271|597
Full-length proteins (EvidentialGene)	86.7%
Transcriptome completeness	C: 79.1% [S: 38.2%, D: 40.9%], F: 4.7%, M: 16.2%, n: 3354

**Table 4:** Overview of the annotation results.

Database	Hits (%)
UniProt/SwissProt Blastx	28,274 (70.7%)
UniProt/SwissProt Blastp	25,051 (62.7%)
Pfam Domains	23,689 (59.2%)
GO	28,399 (71.0%)
KOG	26,409 (66.0%)
KEGG	26,097 (65.3%)
Transmembrane Domains (TmHMM)	7155 (17.9%)
Signal Peptides (SignalP)	2305 (5.8%)
Non-coding RNAs (Infernal)	169 (0.4%)
Non-redundant protein DB (NR Blastx)	33,049 (82.7%)
Non-redundant protein DB (NR Blastp)	28,303 (70.8%)
Nucleotide DB (NT Blastn)	39,827 (99.6%)

## Data Availability

All sequencing reads were obtained from the Sequence Read Archive (SRA) database of the NCBI. The accession numbers can be found in the Materials and Methods section, under ‘Data Selection’. The TrAnnoScope pipeline can be accessed via https://github.com/aysevllpkts/TrAnnoScope (accessed on 27 November 2024).

## Supplementary Materials

The following supporting information can be downloaded at https://www.mdpi.com/article/10.3390/genes15121547/s1, Supplementary File S1: Table S1: Overview of RNA-Seq samples and platforms, Table S2: BUSCO classification of transcript completeness before and after error correction, Table S3: Statistical analysis of error correction effects across BUSCO categories, Table S4: Full-length representation table against zebra finch protein sequences, Figure S1: FastQScreen mapping results across genomes for Illumina reads, Figure S2: BlobTools contamination assessments for PacBio samples, Figure S3: BUSCO values before and after error correction across BUSCO categories, Supplementary File S2: Annotation and taxonomy information for unmapped hits, Supplementary File S3: Folder structure of the TrAnnoScope pipeline, along with the details of SLURM jobs and runtimes for data, Supplementary File S4: Tidied Trinotate annotation results of the zebra finch transcripts.
