# Supplementary material for: TrAnnoScope: A Modular Snakemake Pipeline for Full-Length Transcriptome Analysis and Functional Annotation
Source: Genes (Basel). 2024 Nov 29;15(12):1547. doi: 10.3390/genes15121547 (PMC11727683; doi:10.3390/genes15121547)
Supplement: Supplementary file 1 [file genes-15-01547-s001.zip › genes-3328937-supplementary/Supplementary File S1.pdf]

Table S1. Overview of RNA-Seq samples and platforms

This table lists the zebra finch RNA-Seq samples, their corresponding Sequence Read Archive (SRA) identifiers, and the sequencing platforms used. The samples include paired-end reads from Illumina NextSeq 500 and long-read sequences from PacBio SMRT Sequel.

**Sample**	**SRA**	**Platform**
5_testis	SRR8551567	Illumina NextSeq 500 (PE 2x76bp)
2_ovary	SRR8551565	Illumina NextSeq 500 (PE 2x76bp)
5_brain	SRR8551563	Illumina NextSeq 500 (PE 2x76bp)
2_brain	SRR8551559	Illumina NextSeq 500 (PE 2x76bp)
5_testis	SRR8551566	PacBio SMRT Sequel
2_ovary	SRR8551564	PacBio SMRT Sequel
5_brain	SRR8551562	PacBio SMRT Sequel
2_brain	SRR8551558	PacBio SMRT Sequel

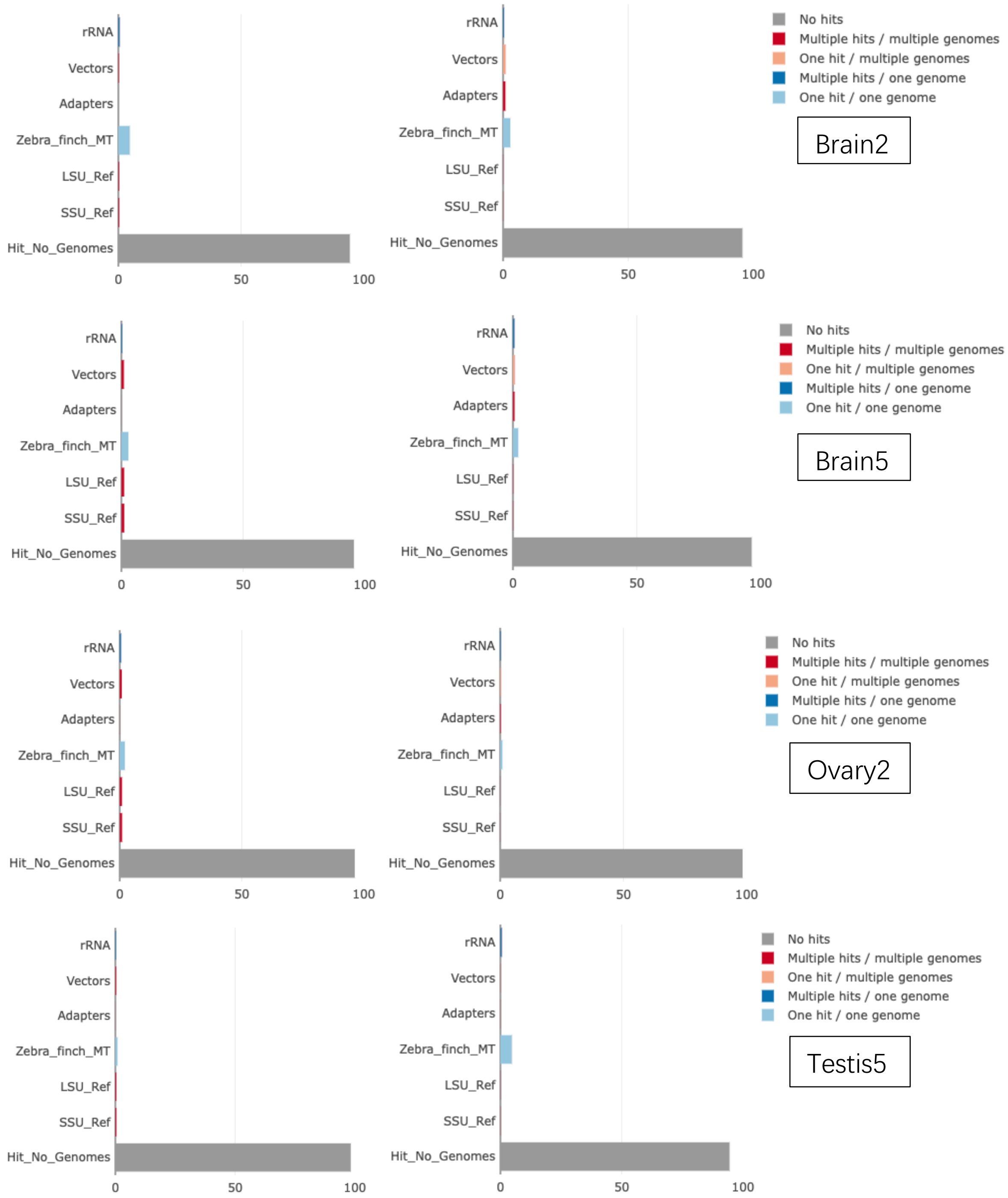

Figure S1. FastQScreen mapping results across genomes for Illumina reads.
This graph displays histograms generated by FastQ Screen for each sample, illustrating the proportion of reads that align to various reference genomes. Each bar in the histogram represents the percentage of reads categorized as uniquely mapped, multiple mapped, or unmapped to specific reference databases (human rRNA, vectors, adapters, zebra finch MT, LSU and SSU). These histograms provide an overview of the sample composition, enabling the detection of contamination and the assessment of the alignment specificity across different reference genomes.

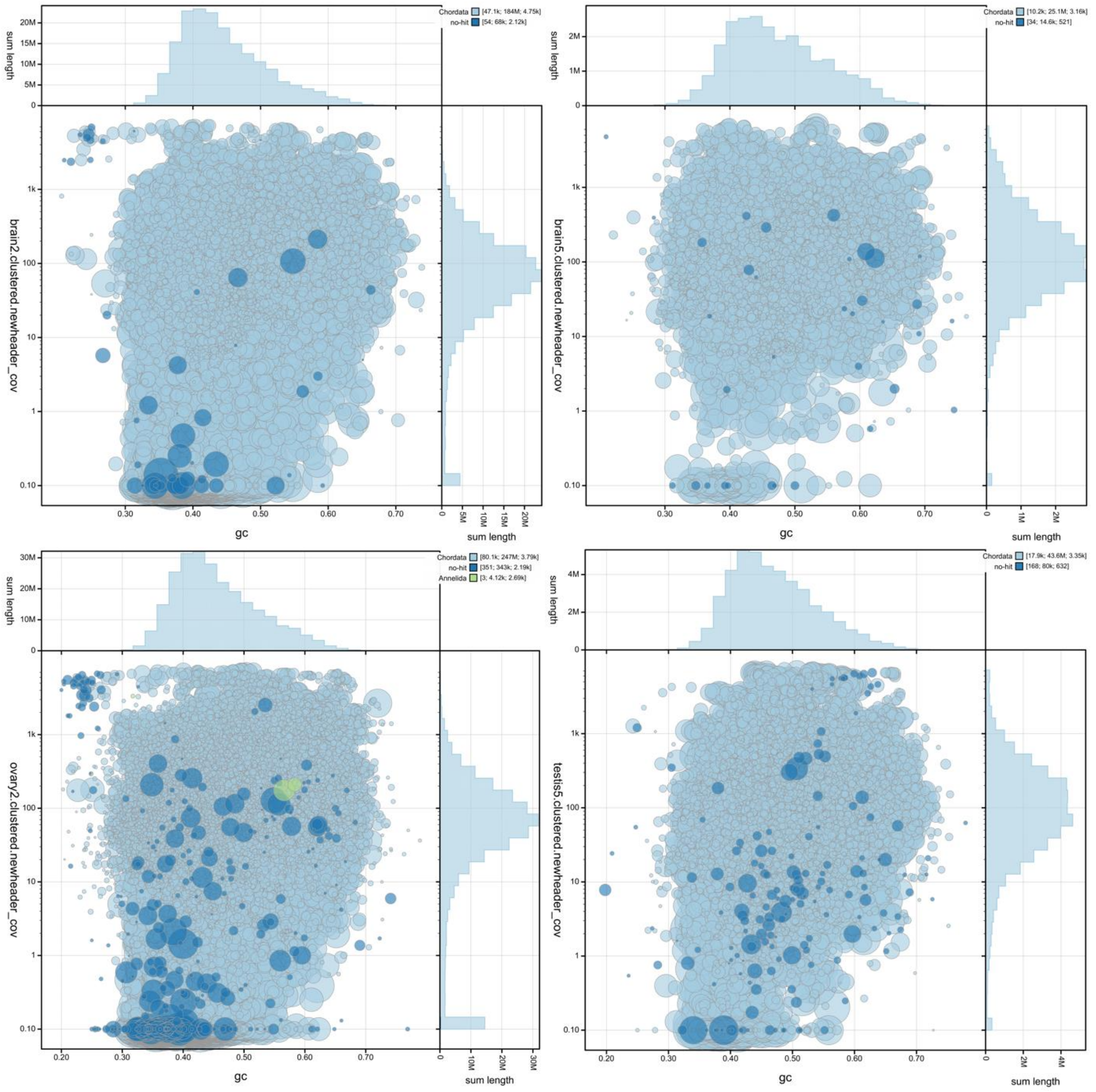

Figure S2. BlobTools2 contamination assessments for PacBio samples.
This graph presents the GC content versus coverage data for each sample processed through the pipeline, with each data point representing a contig or scaffold from the assembly. The x-axis shows the GC content percentage, while the y-axis indicates the sequencing coverage (depth). Points are color-coded based on their taxonomic classification, allowing for the identification of distinct clusters corresponding to different organisms or potential contaminants. This combined view helps in assessing assembly quality, detecting contamination, and distinguishing between sequences of different biological origins across all samples.

Table S2. BUSCO classification of transcript completeness before and after error correction.

This table shows the number of complete, single, duplicated, fragmented, and missing BUSCOs for four tissues (Brain2, Brain5, Ovary2, and Testis5) before and after error correction. The data highlight changes in transcript classification, with increases in complete and duplicated BUSCOs and decreases in single, fragmented and missing BUSCOs following error correction.

	Before error correction	After error correction
	**Brain2**	
**Complete**	1785	1900
**Single**	916	644
**Duplicated**	869	1256
**Fragmented**	212	159
**Missing**	1357	1295
	**Brain5**	
**Complete**	852	993
**Single**	676	613
**Duplicated**	176	380
**Fragmented**	168	104
**Missing**	2334	2180
	**Ovary2**	
**Complete**	2180	2315
**Single**	842	607
**Duplicated**	1338	1708
**Fragmented**	264	204
**Missing**	910	835
	**Testis5**	
**Complete**	1364	1548
**Single**	966	748
**Duplicated**	398	800
**Fragmented**	256	149
**Missing**	1734	1657

Table S3. Statistical analysis of error correction effects across BUSCO categories. This table presents the p-values from Shapiro-Wilk tests for normality and paired t-tests comparing the before error correction (bEC) and after error correction (aEC) for BUSCO categories: complete, single, duplicated, fragmented, and missing. The paired t-test p-values indicate the statistical significance of the differences between bEC and aEC for each category.

**Category**	**Shapiro bEC p-value**	**Shapiro aEC p-value**	**Test type**	**p-value**
complete	0.9659	0.9782	Paired t-test	0.0022
single	0.5944	0.1263	Paired t-test	0.0235
duplicated	0.7773	0.9629	Paired t-test	0.0051
fragmented	0.4854	0.9086	Paired t-test	0.0101
missing	0.9778	0.9829	Paired t-test	0.0003

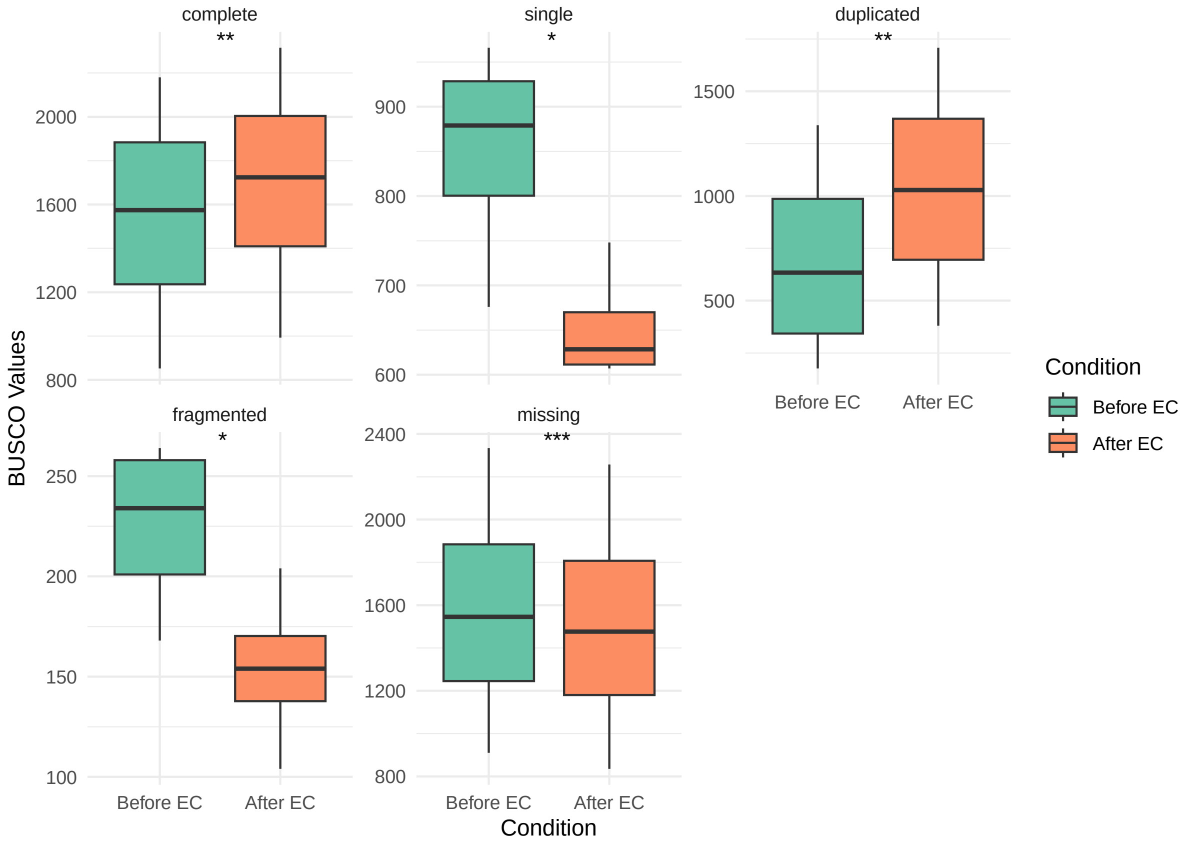

Figure S3. BUSCO values before and after error correction across different BUSCO categories.

The plot shows the distribution of BUSCO values (complete, single, duplicated, fragmented, and missing) before and after error correction (EC). Data is presented as boxplots, with each panel corresponding to a different BUSCO category. Statistical significance between conditions (before and after EC) is indicated by asterisks (*). The analysis was performed using a paired t-test.

Table S4. Full-length representation table against zebra finch protein sequences. Distribution of transcript coverage percentages against zebra finch protein sequences (GCF_003957565.2) from Diamond BLASTP with e-value of 1e-20. The table lists the number of transcripts within each coverage bin and the cumulative total, reflecting the completeness of assembly. Each bin represents a range of coverage percentages, with cumulative count indicating the total number of transcripts meeting or exceeding each bin's lower bound.

**perc_cov_bin**	**count_in_bin**	**>cumulative_bin**
[100-90)	21382	21382
[90-80)	2162	23544
[80-70)	1016	24560
[70-60)	599	25159
[60-50)	353	25512
[50-40)	208	25720
[40-30)	176	25896
[30-20)	177	26073
[20-10)	65	26138
[10-0)	3	26141
