# Supplementary material for: TrAnnoScope: A Modular Snakemake Pipeline for Full-Length Transcriptome Analysis and Functional Annotation
Source: Genes (Basel). 2024 Nov 29;15(12):1547. doi: 10.3390/genes15121547 (PMC11727683; doi:10.3390/genes15121547)
Supplement: Supplementary file 1 [file genes-15-01547-s001.zip › genes-3328937-supplementary/Supplementary File S3/Supplementary File S3.pdf]

Supplementary File S3. Folder structure of the TrAnnoScope pipeline

This supplementary file provides an overview of the directory structure generated by the TrAnnoScope pipeline, detailing the outputs from each stage of the workflow.

(B) Quality Control

```
├── illumina/
│   ├── qc/
│   │   ├── rawFastQC/
│   │   └── rawMultiQC/
```

(E) Illumina Preprocessing

```
│   ├── filtered_fastqscreen/
│   ├── trimmed_fastp/
│   │   ├── brain2_1.trimmed.fq.gz
│   │   ├── brain2_2.trimmed.fq.gz
│   │   ├── brain2.html
│   │   ├── brain2.json
│   │   ├── brain5_1.trimmed.fq.gz
│   │   ├── brain5_2.trimmed.fq.gz
│   │   ├── brain5.html
│   │   ├── brain5.json
│   │   ├── ovary2_1.trimmed.fq.gz
│   │   ├── ovary2_2.trimmed.fq.gz
│   │   ├── ovary2.html
│   │   ├── ovary2.json
│   │   ├── testis5_1.trimmed.fq.gz
│   │   ├── testis5_2.trimmed.fq.gz
│   │   ├── testis5.html
│   │   └── testis5.json
│   ├── trimmedFastQC/
│   └── trimmedMultiQC/
```

(D) PacBio Preprocessing

```
├── pacbio/
│   ├── ccs/
│   ├── fl/
│   ├── flnc/
│   ├── clustered/
```

(F) Post-processing

```
│   ├── remove_contaminants/
│   │   ├── blastn/
│   │   ├── blobtools/
│   │   ├── busco_clustered/
│   │   ├── clean_reads/
│   │   └── coverage/
│   ├── error_correction/
│   ├── classification/
│   │   ├── cdhit/
│   │   └── evigene/
```

(D) Quality Assessment

```
├── quality_assessment/
│   ├── busco/
│   │   └── vertebrata_odb10_transcriptome/
│   ├── fl_representation/
│   │   ├── against_zebrafinch/
│   │   ├── transcriptome_uniprot_blastp.outfmt6
│   │   ├── transcriptome_uniprot_blastp.outfmt6.coverage.hist
│   │   ├── transcriptome_uniprot_blastx.outfmt6
│   │   └── transcriptome_uniprot_blastx.outfmt6.coverage.hist
│   └── stats/
│       ├── protein_completeness.csv
│       ├── protein_completeness_plot_detailed.png
│       ├── protein_completeness_plot.png
│       ├── transcriptome_nt_NanoPlot-report.html
│       ├── transcriptome_nt_NanoStats.txt
│       ├── transcriptome_nt_Non_weightedHistogramReadlength.{html/png}
│       ├── transcriptome_nt_Non_weightedLogTransformed_HistogramReadlength.{html/png}
│       ├── transcriptome_nt_WeightedHistogramReadlength.html
│       ├── transcriptome_nt_WeightedHistogramReadlength.png
│       ├── transcriptome_nt_WeightedLogTransformed_HistogramReadlength.{html/png}
│       ├── transcriptome_nt_Yield_By_Length.{html/png}
│       ├── transcriptome_prot_dist_plot.png
│       └── transcriptome_prot_summary.txt
│
```

```
└── transcriptome/
│     ├── gene_trans_map.txt
│     ├── split/
│     ├── transcriptome_nt.fasta
│     ├── transcriptome_prot.fasta
│     └── transcriptome_prot_modified.fasta
├── annotation/
│     ├── eggnog_mapper/
│     ├── infernal/
│     ├── nr_blastp/
│     ├── nr_blastx/
│     ├── nt_blastn/
│     ├── pfam/
│     ├── plots/
│     │     ├── zebrafinch_full_ALL_GOSlims.pdf
│     │     ├── zebrafinch_full_Biological_Process_GOSlims.pdf
│     │     ├── zebrafinch_full_Cellular_Component_GOSlims.pdf
│     │     ├── zebrafinch_full_COG_dist.pdf
│     │     ├── zebrafinch_full_KEGG_dist.pdf
│     │     ├── zebrafinch_full_KEGG_slim_dist.pdf
│     │     ├── zebrafinch_full_Molecular_Function_GOSlims.pdf
│     │      ├── zebrafinch_full_top10_species_dist_NR_blastx.pdf
│     ├── signalp6/
│     ├── swissprot_blastp/
│     ├── swissprot_blastx/
│     ├── tmhmm2/
│     ├── zebrafinch_full_trinotate_report_trans.goslims
│     └── zebrafinch_full_trinotate_report.xls
│
```

(G) Annotation

Analysis Details and Runtime

The transcriptome analysis was executed on a SLURM-based high-performance computing cluster. Each step of the Snakemake pipeline was submitted as an independent SLURM job, running in parallel to optimize performance and reduce runtime.

Total runtime: The entire pipeline took approximately 8 hours to complete, with various jobs running in parallel.
SLURM job submission:
Each Snakemake rule was executed as a separate SLURM job.
    Number of jobs: 517
    Resources per job: Resources were defined in the config.yaml file.
SLURM job parameters:
    CPUs per job: defined in the config.yaml file.
    Memory per job: defined in the config.yaml file.
